# The effects of circadian desynchronization on alcohol consumption and affective behavior during alcohol abstinence in female rats

**DOI:** 10.3389/fnbeh.2022.1044783

**Published:** 2022-12-22

**Authors:** Christiane Meyer, Konrad Schoettner, Shimon Amir

**Affiliations:** Department of Psychology, Center for Studies in Behavioral Neurobiology, Concordia University, Montreal, QC, Canada

**Keywords:** alcohol consumption, alcohol abstinence, females, behavioral tests, anxiety, estrous cycle, circadian disruption

## Abstract

Disruption of circadian rhythmicity distorts physiological and psychological processes and has major consequences on health and well-being. A chronic misalignment within the internal time-keeping system modulates alcohol consumption and contributes to stress-related psychiatric disorders which are known to trigger alcohol misuse and relapse. While there is growing evidence of the deleterious impact of circadian disruption on male physiology and behavior, knowledge about the effect in females remains limited. The present study aims to fill the gap by assessing the relationship between internal desynchronization and alcohol intake behavior in female rats. Female Wistar rats kept under standard 24-h, 22-h light-dark conditions, or chronic 6-h advanced phase shifts, were given intermittent access to 20% alcohol followed by an extended alcohol deprivation period. Alcohol consumption under altered light-dark (LD) conditions was assessed and emotional behavior during alcohol abstinence was evaluated. Internally desynchronization in female rats does not affect alcohol consumption but alters scores of emotionality during alcohol abstinence. Changes in affective-like behaviors were accompanied by reduced body weight gain and estrous irregularities under aberrant LD conditions. Our data suggest that internal desynchronization caused by environmental factors is not a major factor contributing to the onset and progression of alcohol abuse, but highlights the need of maintaining circadian hygiene as a supportive remedy during alcohol rehabilitation.

## Introduction

Alcohol abuse is a widespread public health concern with adverse social and economic ramifications. Individuals with alcohol use disorder (stocktickerAUD) have impaired control over drinking and continue to drink despite deleterious consequences to their health and professional lives (Rehm et al., 2009). stocktickerAUD affects over 280 million people worldwide (Glantz et al., 2020), including more than 18% of the Canadian population (Statistics Canada, 2013). Clinical studies indicate that workers with night and rotating working schedules are more likely to suffer from binge drinking disorders compared to daytime workers (reviewed by Richter et al., 2021).

Although the causes of excessive alcohol consumption are multifactorial, including genetic, socio-economical, and psychological factors, growing evidence point to the important role of the internal time-keeping system in the development and progression of alcohol abuse (Parekh et al., 2015; Partonen, 2015; Barko et al., 2019). The circadian system refers to the hierarchical coordination of biological clocks located in the brain and peripheral tissues and organs that control daily rhythms in physiology and behavior. These biological timers rely on so-called clock genes and their protein products to generate 24-h rhythms and their synchronization to environmental periodicities such as the solar cycle. Genetic studies in humans and rodents revealed the influence of circadian clock gene modification on excessive alcohol consumption and abuse (Spanagel et al., 2005; Kovanen et al., 2010). In addition, disruption of circadian rhythms has been shown to alter alcohol consumption, which is associated with differential accumulation of ΔFosB in brain regions related to the control of alcohol consumption (Reséndiz-Flores and Escobar, 2019).

Nevertheless, circadian rhythm disruption affects not only the reward-related neural circuitry but also contributes to stress-related mood disorders. Clinical studies have linked clock gene variants with depressive and anxiety-like behavior in humans (reviewed by Partonen, 2012). Moreover, rodents exposed to constant light or continuous alterations in LD cycles not only increase alcohol consumption but also promote the development of anxiety and depression-like behavior (Clark et al., 2007; Rosenwasser et al., 2010, 2015). Indeed, alcohol misuse is highly comorbid with neuropsychiatric disorders including anxiety and depression, and diagnosed patients have poorer alcohol rehabilitation outcomes, and a higher probability of relapse (Lynskey, 1998; Heilig et al., 2010). The intensity and context of craving to consume alcohol stem from emotional discomfort during alcohol abstinence which may explain an increased relapse risk (Karpyak et al., 2016; Koob and Volkow, 2016).

Although not fully understood, excessive alcohol consumption and recovery may be driven, in part, by sex-specific neurobiological mechanisms (Becker and Koob, 2016; McHugh et al., 2018). While men were more often diagnosed with AUD than women, recent evidence points to a substantial increase in alcohol consumption among women, closing the gender gap in alcohol abuse and dependence (Ait-Daoud et al., 2017; McKetta and Keyes, 2019). AUD-diagnosed women are more likely to display comorbid affective symptoms compared to men (Abulseoud et al., 2013; Holzhauer et al., 2019). In particular, negative emotions are stressful for women, which cues more alcohol reinforcement and craving than in men (Palma-Álvarez et al., 2019; Peltier et al., 2019). Thus, negative affect and stress play a pivotal role in the development of addiction, including initiation and relapse, in particular for women (Koob, 2009).

Even though there is emerging evidence that chronodisruption affects alcohol drinking behavior differently in males and females (Rizk et al., 2022), only little is known about the sex-specific consequences of chronodisruption on mood, and their interaction in the development of AUD. The relationship between circadian rhythm disruption and changes in reward and mood-related behavior remains poorly understood. Besides the utmost importance of the female sex in alcohol studies, research mostly focuses on clinical male cohorts or male rodents, leaving implications for the female sex in doubt (Zucker and Beery, 2010). To address this, we used female rats to investigate the effects of internal desynchrony on binge-drinking-like behavior, and emotion-related behavior during alcohol abstinence.

Our findings demonstrate that internal desynchronization has only minor effects on alcohol-drinking behavior in female rats but amplifies emotionality during alcohol abstinence. This study represents an important step in understanding the effects of circadian disruption on behavioral and physiological processes connected to reward and mood in the female sex.

## Materials and methods

### Animals and housing

Adult female Wistar rats (age 95–120 days, Charles River Laboratories, Saint-Constant, Canada) were kept individually in transparent cages equipped with running wheels (Tecniplast, Buguggiate, VA, Italy) in light (standardized illuminance of ∼200–300 lux during the light period, 0 lux during the dark) and temperature-and humidity controlled (temperature: 21 ± 1°C, relative humidity: 65 ± 5%) rooms. All animals had access to food and water *ad libitum*. Food consumption and body weight were monitored once a week throughout the experiment. Prior to the experiment, rats were kept under a standard 12:12 h light-dark (LD24) schedule for 3 weeks. Following acclimatization, all animals were randomly assigned to be kept under standard (CTRL) or desynchronizing lighting conditions. We used two different light-dark (LD) paradigms to induce circadian desynchrony: A chronic phase shift schedule (SHIFT), consisting of 6-h advanced shifts in the onset of the light phase every second day, by shortening the dark phase (Casiraghi et al., 2012), and a short-day 11:11 h light-dark (LD22) paradigm, shown to induce desynchronization between the two oscillators in the core and shell of the suprachiasmatic nucleus (SCN) (de La Iglesia et al., 2004).

### Alcohol drinking paradigm

To induce voluntary alcohol consumption in rats, the intermittent alcohol exposure (IAE20%) paradigm was used (Wise, 1973). The IAE20% drinking paradigm resulted in an efficacious model for inducing high levels of voluntary alcohol consumption in several rat strains (Wise, 1973; Simms et al., 2008; Mill et al., 2013). The protocol represents an alcohol binge-drinking-like pattern by alternating alcohol access and deprivation periods. Alcohol-naïve rats were given access to 20% ethanol solution (v/v) and tap water (two-bottle choice) for three 24-h sessions per week (Monday, Wednesday, and Friday). On alcohol-free days (Tuesdays, Thursdays, Saturdays, and Sundays), rats received water only. Fluid bottles were weighed and replaced daily at ZT4 (ZT- Zeitgeber time; ZT0 represents the time of light onset). Bottles were weighed before and after the replacement in all groups. To control for side preferences, the placement of the bottles was alternated during the alcohol-drinking session.

### Behavioral tests

#### Sucrose preference test (SPT)

Reward-based anhedonia-like behavior was assessed by monitoring sucrose intake (Eagle et al., 2016). First, rats were habituated to two bottles of 1% sucrose solution (m/v) for 48 h to avoid neophobia. Subsequently, animals underwent food and water deprivation for 20 h. In the final SPT, all animals had access to one bottle of 1% sucrose solution and one bottle of water for 3 h. The total amount of sucrose solution and water intake during this period was recorded. All groups were tested at ZT2. Sucrose preference was calculated as a percentage of the volume of sucrose intake over the total volume of fluid intake.

#### Open field test (OFT)

The OFT was performed to evaluate exploratory and anxiety-like behavior (Prut and Belzung, 2003). Prior to the test, animals were habituated to the testing room for 30 min. At the beginning of the test, each rat was placed at the left-back corner of the open field arena (39 × 42 × 50 cm, TruScan Photo beam Activity Monitors, Coulbourn Instruments, Holliston, USA). The animals were allowed to freely explore the arena for 5 min. The animals’ location and movements were recorded by infrared beams and analyzed by the software TruScan 2.0 (Coulbourn Instruments, Holliston, USA). All animals were tested at ZT2.

#### Elevated plus-maze (EPM)

The EPM was used to assess anxiety-related behavior (Walf and Frye, 2007). The maze consists of two open arms and two closed arms (30 × 10 cm, respectively), elevated 50 cm above the floor. The test was performed at the beginning of the dark phase, 2 h after the lights were turned off (ZT14). All animals were habituated to the room conditions for 30 min. To start the test, rats were placed in the middle of the intersection between the open and closed arms facing the open arms. Behavior was video-recorded for 5 min.

#### Marble burying task (MBT)

The MBT was used to evaluate impulsiveness, irritability, and compulsive behavior. Burying behavior of harmless objects such as marbles is suggested as a form of impulsive behavior (Schneider and Popik, 2007) or indicates high-anxiety levels (Li et al., 2019). Twenty glass marbles (ø 1.5 cm) of different colors were evenly spaced in five rows of four in a transparent plexiglass cage measuring 34 × 54 cm, filled with Sani Chips bedding (Teklad Envigo, Costa Mesa, USA). At ZT2, rats were placed individually in the cages and were allowed to explore the cages for 30 min. The number of buried marbles was counted at the end of the 30 min period. Marbles were considered buried if two-thirds of the marble was covered with bedding.

### Experimental procedures

#### Experiment 1–Effect of different LD schedules and alcohol consumption on circadian disruption

**FIGURE 1:**
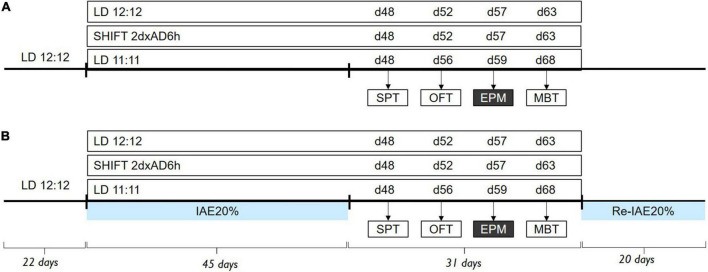
Experimental design. Timeline of alcohol exposure and behavioral assessment **(A)** under LD24 (*n* = 12), SHIFT (*n* = 6), LD22 (*n* = 4) conditions, and **(B)** with access to intermittent alcohol exposure (LD24 *n* = 12, SHIFT *n* = 12, LD22 *n* = 12). Behavior tests were conducted during the light phase (ZT2), except for the EPM (ZT14). Zeitgeber (ZT) indicates hours after the onset of the light phase.

In the first experiment, we evaluated the impact of various light conditions and alcohol consumption on circadian rhythmicity (Figure 1). Internal desynchronization was assessed by recording the locomotor activity under LD24 (*n* = 12), SHIFT (*n* = 6), and LD22 (*n* = 4) independently and in combination with alcohol consumption, deprivation, and re-introduction of alcohol (LD24 *n* = 12; SHIFT *n* = 12; LD22 *n* = 12). The severity of chronodisruption was assessed using the following circadian parameters: daily fragmentation (intradaily variability–IV), day-to-day stability (interdaily stability–IS), and the difference between day and night activity levels (relative amplitude–RA).

#### Experiment 2–Effect of different LD schedules on alcohol consumption

This experiment determines if rats kept under artificial LD conditions will voluntarily consume more alcohol in a two-bottle choice paradigm compared to animals under standard LD24 conditions. Animals were kept either under LD24, SHIFT, or LD22 conditions and given intermittent access to alcohol for 20 sessions (Figure 1B). We assessed alcohol preference (%) over water and alcohol intake (g/kg/24 h), as well as the relationship between alcohol preference and the animals’ circadian rhythmicity under alcohol exposure.

Following alcohol access, alcohol was withheld to determine if high alcohol consumption levels would be maintained following a prolonged abstinence period. Thereafter, the animals were given again intermittent access to 20% alcohol for 10 alcohol-drinking sessions as described above. Comparisons were made between the mean alcohol consumption and alcohol preference, respectively, during the 10 alcohol-drinking sessions immediately before and after the abstinence period.

#### Experiment 3–Effect of different LD schedules on emotional behavior during alcohol abstinence

We next compared the behavioral performance of animals kept under LD alterations alone and LD alterations during alcohol abstinence in a variety of behavioral tests (Figure 1). In the alcohol groups, behavioral tests were conducted 24 h after the last alcohol access, under alcohol-free conditions. Animals followed the same testing order, starting with SPT, followed by the OFT, EPM, and MBT. The differences in the testing days of SHIFT and LD22 are due to the external LD schedule to test animals at the same zeitgeber time. Animals had a period of three to 5 days to recover between tests.

#### Experiment 4–Effect of different LD schedules during alcohol access and abstinence on body weight and estrous regularity

We determined the effect of chronic LD alterations on body weight without and with alcohol access and deprivation. Relative changes in body weight were calculated based on the individual body weight at the beginning of the experiment. As stress and artificial light conditions influence the female estrous regularity, we characterized the estrus cycle over 24 days (24 days as a multiplicand of a 4-day estrous cycle) of each LD paradigm and alcohol condition.

### Data analysis

We quantified circadian rhythmicity by continuous registration of locomotor activity using running wheels. Summed wheel revolutions were recorded and stored over 10 min intervals using the VitalView system (Mini-Mitter, Starr Life Sciences Corp., Oakmont, USA). Locomotor activity data from the last 14 days of each LD condition and phase of alcohol exposure were used for the Chi-square-periodogram and circadian parameter analysis. Chi-square-periodograms (Sokolove and Bushell, 1978; Refinetti, 1992) were compiled to estimate the period of the daily activity rhythms under the various LD conditions (LD24, SHIFT, LD22) and in combination with alcohol access and abstinence using ClockLab 6 (Actimetrics Software, Evanston, United States). Results were reported as Chi-square amplitude. To obtain additional measures of activity disruption we assessed RA, IV, and IS based on the equations reviewed by Brown et al. (2019) using ClockLab 6 (Actimetrics Software, Evanston, United States) for the last 14 days of each LD and alcohol accessibility phase. Wheel running activities were visualized as double-plotted actograms using ClockLab 6 (Actimetrics Software, Evanston, United States).

Statistical analysis was performed using the GraphPad Prism 9 software package (GraphPad, San Diego, USA). All results were presented as mean ± standard error of the mean (SEM) and were examined for normality and homogeneity of variance. The significance level was set at *p* < 0.05. A one-way ANOVA (LD effect) was used to compare the rhythm parameters IV, IS and RA between the LD regimens, followed by a Dunnett’s *post-hoc* test if a significant main effect was found. Further evaluation of the effect of the light regimen or alcohol exposure on circadian parameters during periods of alcohol access or abstinence was conducted by a two-way ANOVA (LD, alcohol effect) and Dunnett’s *post hoc* comparison.

Alcohol consumption and preference, as well as water consumption between the animals kept under different LD conditions over the 20 exposure sessions, were assessed by a two-way repeated measures ANOVA (LD and time effect). The effect of LD conditions and progression of alcohol exposure on alcohol intake and preference before and after alcohol abstinence was assessed in a two-way repeated measure ANOVA.

Behavioral tests were analyzed using two-way ANOVA (LD effect, alcohol effect), followed by Sidak’s *post hoc* analysis when a significant overall effect was found.

A two-way ANOVA was conducted to evaluate the effect of the different LD conditions and alcohol exposure on body weight. The female estrous cyclicity was assessed by analyzing wheel running activity (Wollnik and Turek, 1988) over a period of 24 days within each alcohol availability phase under standard or altered LD conditions by chi-square analysis using the LSP software^1^ (Sokolove and Bushell, 1978). This method provides a longitudinal, and non-invasive approach to avoid the risk of false-positive/negative results by inducing an irregular estrous cycle or the status of pseudopregnancy compared to the vaginal smears method (Cora et al., 2015). We characterized the estrous cyclicity by the number of rats showing a regular estrous cycle and evaluated the effects of LD conditions or alcohol using a two-way ANOVA.

## Results

### Experiment 1–Effect of different LD schedules and alcohol consumption on circadian disruption

**FIGURE 2:**
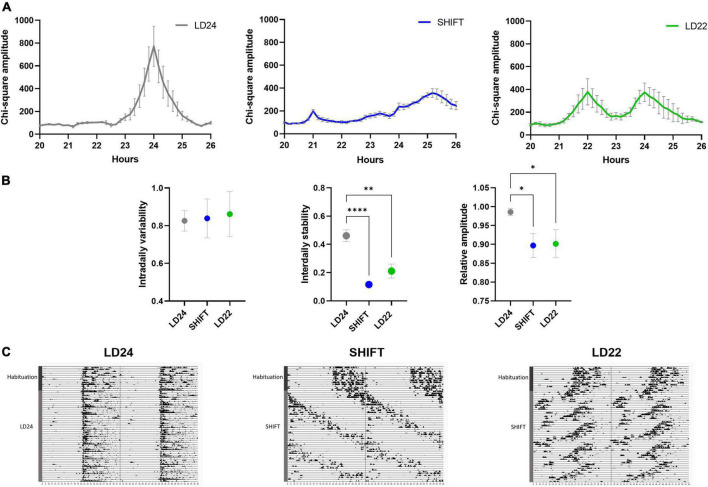
Locomotor activity under altered light-dark (LD) schedules. **(A)** Comparison of periodograms and actograms under LD24, SHIFT, and LD22 conditions and **(B)** determination of intradaily variability (IV), interdaily stability (IS), and relative amplitude (RA) over a period of 14 days. One-way ANOVA, Dunnett’s multiple comparisons test: ****p < 0.0001, **p < 0.01, *p < 0.05; ± SEM; *n* = 4–12. **(C)** Representative circadian actograms for one animal for each LD paradigm (50 days). Time of day is double-plotted along the x-axis, and successive days are arranged from top to bottom along the y-axis.

**FIGURE 3:**
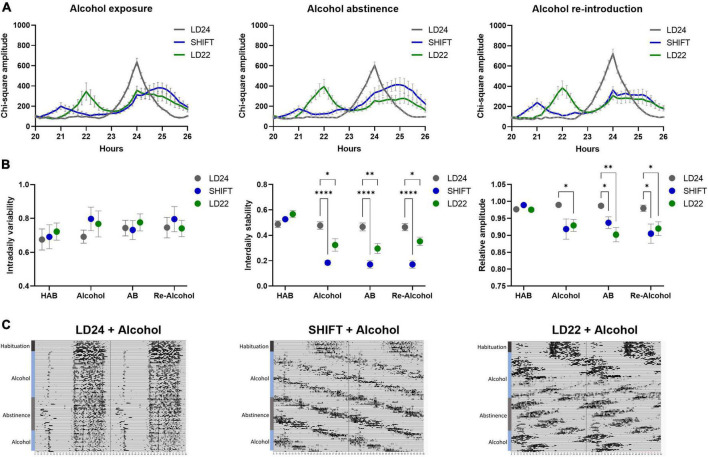
Locomotor activity under altered light-dark (LD) conditions and intermittent alcohol exposure. **(A)** Comparison of periodograms and actograms during access to alcohol, alcohol abstinence, and re-introduction of alcohol. **(B)** Intradaily variability (IV), interdaily stability (IS), and relative amplitude (RA) during habituation (HAB), intermittent access to 20% alcohol solution (Alcohol), abstinence (AB), and reintroduction of alcohol after abstinence (Re–Alcohol). Two-way ANOVA, Dunnett’s multiple comparisons test: ****p < 0.0001, **p < 0.01, *p < 0.05; ± SEM; *n* = 4–12/LD condition. **(C)** Representative circadian actograms for one animal for each LD paradigm and alcohol exposure. Time of day is double-plotted along the x-axis, and successive days are arranged from top to bottom along the y-axis and labeled with the coinciding alcohol exposure phase.

Exposure to non-24-h LD cycles had a significant effect on daily activity rhythms (Figure 2). Internal desynchronization was observed in animals kept under SHIFT and LD22, indicated by the occurrence of two distinct activity peaks (Figure 2A). Assessment of various rhythm parameters confirmed the deleterious effect of non-24 h LD conditions on daily locomotor activity patterns (Figure 2B). Whereas IV was not affected by the LD conditions [F_(2_,_17)_ = 0.04154, *p* = 0.9594, one-way ANOVA], a significant main effect of the LD conditions on IS [F_(2_, _17)_ = 21.85, *p* < 0.0001, one-way ANOVA] and RA [F_(2_,_17)_ = 5.845, *p* = 0.0117, one-way ANOVA] was found. Both parameters were significantly reduced in animals exposed to non-24-h light cycles (Figure 2B). When considering alcohol exposure as an additional factor besides the LD condition, the same rhythm parameters as mentioned above were significantly affected by both factors (Figures 3A, B, Table 1). Interestingly, the alcohol effect on IS and RA was primarily accompanied by the change in the LD condition, and the significant interaction underpinned this conclusion. In LD24 animals, there was no effect of the alcohol condition on the rhythm stability throughout the experiment (Figure 3B). Moreover, no differences in IS and RA between alcohol exposure vs alcohol-free periods under chronically altered LD conditions (SHIFT or LD22) were found (Figure 3B).

**TABLE 1:** Statistical results of circadian parameters for the combination of altered light-dark (LD) schedules and alcohol consumption.

Parameter	Two-way ANOVA		
	LD condition	Alcohol condition	LD × Alcohol
IV	F_(2, 33)_ = 0.3046, *p* = 0.7792.	F_(2_._798, 92_._34)_ = 1.197, *p* = 0.2987	F_(6, 99)_ = 0.5810, *p* = 0.8264
IS	F_(2, 33)_ = 24.37, *p* < 0.0001	F_(2_._769, 91_._38)_ = 42.75, *p* < 0.0001	F_(6, 99)_ = 10.93, *p* < 0.0001
RA	F_(2, 33)_ = 7.754, *p* = 0.0017	F_(2_._729, 90_._05)_ = 5.378, *p* = 0.0026	F_(6, 99)_ = 2.479, *p* = 0.0282

### Experiment 2–Effect of different LD schedules on alcohol consumption

#### Experiment 2a–Effect of different LD schedules on intermittent alcohol consumption

**FIGURE 4:**
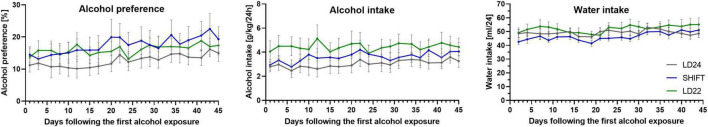
Time course for alcohol and water consumption in rats exposed to LD24, SHIFT, and LD22 paradigms. ± SEM; *n* = 12/LD paradigm.

No significant effect of the LD condition on alcohol intake, preference, and water intake was found (Figure 4, Table 2). A significant effect of the progression of the experiment on alcohol preference was observed, indicating that animals’ preference for alcohol increases over time, irrespective of the LD conditions.

**TABLE 2:** Statistical results of fluid consumption and the abstinence effect on fluid consumption.

Parameter	Two-way repeated measures ANOVA		
	LD condition	Time	LD × Time
Alcohol preference	F_(2, 33)_ = 0.8352, *p* = 0.4428	F_(6_._717, 221_._7)_ = 2.883, *p* = 0.0075	F_(38, 627)_ = 0.5337, *p* = 0.9907
Alcohol intake	F_(2, 33)_ = 1.463, *p* = 0.2461	F_(6_._656, 219_._6)_ = 1.170, *p* = 0.3219	F_(38, 627)_ = 0.4835, *p* = 0.9965
Water intake	F_(2, 33)_ = 1.276, *p* = 0.2926	F_(2_._051, 67_._68)_ = 2.820, *p* = 0.0653	F_(36, 594)_ = 0.9108, *p* = 0.6206
Abstinence effect: Alcohol preference	F_(2, 33)_ = 0.5746, *p* = 0.5684	F_(1, 33)_ = 3.948, *p* = 0.0553	F_(2, 33)_ = 0.1279, *p* = 0.8803
Abstinence effect: Alcohol intake	F_(2, 33)_ = 1.086, *p* = 0.3494	F_(1, 33)_ = 2.178, *p* = 0.1495	F_(2, 33)_ = 0.01568, *p* = 0.9844
Abstinence effect: Water intake	F_(2, 33)_ = 0.3169, *p* = 0.7306	F_(1, 33)_ = 0.08374, *p* = 0.7741	F_(2, 33)_ = 2.169, *p* = 0.1304

#### Experiment 2b–Effect of different LD schedules on a prolonged period of abstinence and subsequent alcohol consumption

After 20 sessions of voluntary alcohol consumption, animals from Experiment 2a were deprived of alcohol and re-exposed to alcohol after 31 days of abstinence. The LD conditions did not affect alcohol re-exposure, and no effect was found on either alcohol intake or preference. We did not observe an effect of sessions on alcohol drinking or preference and water consumption over 10 drinking sessions before and after prolonged alcohol abstinence either (Table 2).

### Experiment 3–Effect of different LD schedules on emotional behavior during alcohol abstinence

**FIGURE 5:**
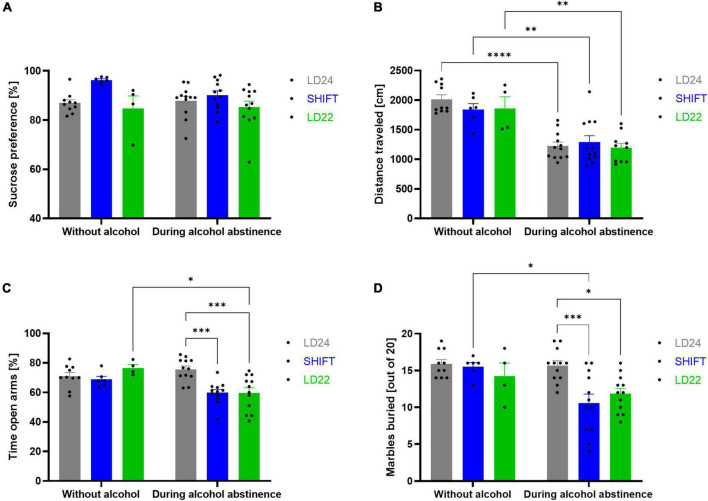
Effect of light-dark (LD) schedule and alcohol abstinence on behavioral performance. **(A)** Sucrose preference (%) over a 3-h test period, **(B)** distance traveled in the OFT, **(C)** the percentage of time spent in the open arms during the EPM, and **(D)** marbles buried within the MBT. One-way ANOVA, Tukey’s multiple comparisons test: ****p < 0.0001, ***p < 0.001, **p < 0.01, *p < 0.05; ± SEM; *n* = 4–12/LD condition.

Emotional and reward-related behaviors were evaluated in animals exposed to standard (LD24) and artificial LD paradigms (SHIFT, LD22) during alcohol abstinence (Figure 5).

### Sucrose preference test

A significant main effect of the LD conditions was found (Figure 5A, Table 3), indicating that sucrose preference was altered under aberrant LD conditions. Subsequent *post hoc* analysis, however, did not reveal significant differences between groups.

**TABLE 3:** Statistical results of behavioral performances.

Parameter	Two-way repeated measures ANOVA		
	LD condition	Alcohol condition	LD × Alcohol
Sucrose preference (SPT)	F_(2, 50)_ = 6.269, *p* = 0.0037	F_(1_,_50)_ = 0.6940, *p* = 0.4088	F_(2_,_50)_ = 1.518, *p* = 0.2292
Distance traveled (OFT)	F_(2, 48)_ = 0.4203, *p* = 0.6592	F_(1_,_48)_ = 62.21, *p* < 0.0001	F_(2, 48)_ = 0.8106, *p* = 0.4506
Time open arms (EPM)	F_(2, 50)_ = 5.383, *p* = 0.0067	F_(1_,_50)_ = 8.233, *p* = 0.0060	F_(2_,_50)_ = 6.910, *p* = 0.0022
Marbles buried (MBT)	F_(2, 50)_ = 5.843, *p* = 0.0052	F_(1_,_50)_ = 9.468, *p* = 0.0034	F_(2_,_50)_ = 3.214, *p* = 0.0486

### Open field test

Rats show a strongly reduced exploratory activity in a novel environment during alcohol abstinence, irrespective of the environmental LD conditions. A significant main effect of the alcohol condition on the total distance traveled in the open field was found (Table 3). *Post hoc* analysis revealed a significant decrease in locomotion during alcohol abstinence in all three experimental groups (Figure 5B).

### Elevated plus maze test

Desynchronizing light conditions affected anxiety-like behavior during abstinence in female rats. A significant main effect was found for both, LD and alcohol conditions (Table 3). The significant LD x alcohol interaction, however, indicates that the effect of the alcohol condition was not uniform across the different LD conditions (Figure 5C, Table 3). Anxiety-like behavior is significantly increased during abstinence in rats kept under aberrant LD conditions when compared to rats kept under regular LD24 cycles (Figure 5C).

### Marble burying task

Similar to outcomes in the elevated plus maze, significant main effects for LD, and alcohol condition, as well as a significant interaction between both factors were found in the marble burying test (Figure 5D, Table 3). A significant reduction in the amount of buried marbles was found in rats kept under LD22 and SHIFT cycles compared to LD24 controls during abstinence-only (Figure 5D).

### Experiment 4–Effect of different LD schedules during alcohol access and abstinence on body weight and estrous regularity

**FIGURE 6:**
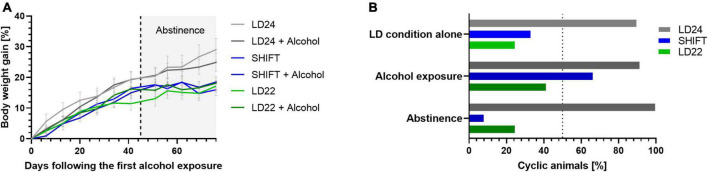
Body weight gain and estrous cyclicity under altered light-dark (LD) schedules, alcohol exposure, and abstinence. **(A)** Change in body weight gain (%) over time. **(B)** Comparison of estrous cyclicity between LD alone and in combination with alcohol access and abstinence as indicated by the percentage of animals showing a rhythmic estrous cycle (%). ± SEM; *n* = 4–12/LD condition.

All rats showed a progressive increase in body weight over time (Figure 6A). Body weight gain (%) differed significantly between the groups depending on time and time x LD paradigm interaction, but not LD condition (Table 4). We also found a pronounced LD effect on the estrous cycle [Figure 6B; LD effect F_(2_, _4)_ = 12.95, *p* = 0.0179, two-way ANOVA]. In contrast, chronic intermittent alcohol consumption alone was not altering the estrous cyclicity under standard LD conditions [Alcohol effect: F_(2_, _4)_ = 1.430, *p* = 0.3399, two-way ANOVA].

**TABLE 4:** Statistical results of body weight gain.

Parameter	Two-way repeated measures ANOVA		
	LD condition	Time	LD × Time
LD alteration	F_(2, 17)_ = 1.784, *p* = 0.1980	F_(2_._417, 41_._08)_ = 70.85, *p* < 0.0001	F_(22, 187)_ = 3.416, *p* < 0.0001
LD alteration + alcohol	F_(2, 33)_ = 2.174, *p* = 0.1298	F_(2_._945, 97_._17)_ = 109.3, *p* < 0.0001	F_(22, 363)_ = 1.477, *p* = 0.0780

## Discussion

The link between the disruptions of the circadian system and alcohol consumption has been recognized for decades. While most of the research focused on male cohorts, the effect of internal rhythm disruption on alcohol consumption in females is less known. We show that the induced internal desynchrony through chronic advanced shifts and LD22 persists throughout the course of alcohol intake and abstinence, but is unaffected by the respective alcohol condition. Although aberrant light schedules, which simulate shiftwork and jet lag, tend to increase alcohol consumption and preference, we observe no significant differences between our experimental groups in female rats. Importantly, we demonstrated that internal desynchrony affects performance in tests of anxiety-like behavior during periods of alcohol absence. However, these behavioral alterations do not seem to predict or interact with alcohol consumption before abstinence or during re-exposure (data not shown). Taken together, our results demonstrate that internal circadian desynchronization strongly affects female physiology, but has only minor effects on mood-related behavior and alcohol consumption if the two factors were considered separately. When combining the effects of alcohol consumption and circadian disruption, changes in mood-related behaviors became more apparent, which emphasizes the important contribution of dysregulation of circadian functioning in the etiology of mood-related behavior in females under alcohol abstinence.

### Alcohol intake under different LD schedules

Experimental conditions of jet lag or constant, abnormal light conditions on alcohol consumption led to disparate results, depending on heterogeneity and breadth in approaches and concepts associated with circadian disruption. In our experiment, we observed similar alcohol intake (g/kg/24 h) levels as previously reported in female Fischer, Lewis rats, and P rats (Rosenwasser et al., 2010, 2014) under standard LD conditions. Although the number of studies on chronodisruption in female rodent models is sparse, previous studies reported conflicting outcomes of chronodisruption on alcohol consumption in female rats (Clark et al., 2007; Rosenwasser et al., 2010). Differences in the alcohol availability paradigm (continuous vs intermittent), alcohol concentration, and LD conditions may account for the different outcomes. Our results, however, are in line with studies conducted in mice that did not reveal differences in alcohol intake or preference in wild-type females exposed to an LD20 cycle compared to animals kept under standard LD conditions (Rizk et al., 2022). Overall, these outcomes indicate that aberrant LD conditions may not be a major factor contributing to abnormal alcohol-drinking behavior in female rodent models.

Numerous factors including species, sex, duration of aberrant light exposure, the direction of phase shift, and the time between shifts affect the independent and combined effects on behavioral and physiological variables (Vetter, 2018). However, studies often lack the quantification and validation of the success of internal rhythm disruption (Brown et al., 2019). In our study, we confirmed that the applied protocols of phase advances and LD22 conditions induce internal desynchrony in female rats, similarly as described previously in male rats (Casiraghi et al., 2012; Ben-Hamo et al., 2016). The degree of internal rhythm disruption, however, was not a strong predictor for alcohol consumption or preference (data not shown). Vice versa, alcohol consumption had no effect on circadian locomotor activity rhythms in female rats investigated in our study, which may be attributed to the use of running wheels. It is known from previous studies in rodents that running wheel activity affects the circadian system (Leise et al., 2013; Weinert et al., 2016; Weinert and Gubin, 2022) whereas contradicting effects were found on alcohol drinking behavior in rodent models, depending on species, sex, and drinking paradigm (Ozburn et al., 2008; Ehringer et al., 2009; Piza-Palma et al., 2014; Buhr et al., 2021). It is, however, conceivable that the independent effects of the running wheel on the circadian system and/or alcohol drinking behavior mitigated the deleterious effects of the aberrant LD conditions and thus, affect alcohol intake and preference. The sex-specific effect of exercise on alcohol drinking should therefore be emphasized in future work.

Several studies indicate that molecular components of the circadian clock play a crucial role in the control of alcohol-drinking behavior (Spanagel et al., 2005; Ozburn et al., 2013) and suggest that the impact of genetic components may even prevail over environmental influences (Rizk et al., 2022). However, the effects of a targeted deletion or downregulation of clock genes are not directly comparable to the effects of chronodisruption. Latter one leaves the clock genes functionally intact but dysregulates in their daily expression, which has been demonstrated in brain areas associated with reward regulation and drug-related behaviors (Tamura et al., 2021). Nevertheless, core clock genes are extensively involved in regulating the dopaminergic reward circuitry and disruption of those processes may increase the vulnerability to developing drug addiction (for review Depoy et al., 2017). Since dopamine transporters and norepinephrine receptors are expressed rhythmically, chronodisruption may dysregulate circadian patterns of neurotransmission and thus alters alcohol exposure (Sleipness et al., 2007). The exact role of circadian clock gene expression in brain regions associated with reward-related processes in female rodents exposed to aberrant LD conditions, however, should be investigated in follow-up studies.

Lastly, peripheral oscillators may impact the reward circuit in addition to the central clock. The release of sex hormones like estradiol is controlled by the circadian system (Barbacka-Surowiak et al., 2003) and the loss of internal rhythmicity affects the pulsatile hormone release (Sciarra et al., 2020). Importantly, sex hormones modulate neuronal activity and can influence alcohol-drinking behavior (Erol et al., 2019). It was shown that the artificial administration of estrogen increases alcohol intake and drug-seeking behaviors in females and males, although fluctuating sex hormone levels during the course of the estrous cycle do not markedly affect alcohol intake (Priddy et al., 2017). In our experiment, internally desynchronized animals show impaired estrous regularity, which may impose altered levels of sex hormones. Altered LD schedules may not only affect the sex hormone function directly but may also act as a chronobiological stressor (Boulos and Rosenwasser, 2004). It is known that stress can affect estrous regularity with consequences on hormone levels including corticosterone and estradiol as well as on the expression of sex hormone receptors (An et al., 2020). Likewise, excessive alcohol consumption disrupts the menstrual cycle in women (Lyngsø et al., 2014) and the estrous cycle in animals (Emanuele et al., 2002). However, similar to Satta et al. (2018), we did not observe an effect of alcohol alone on the estrous cycle regularity in our study. Only in the combination of alcohol consumption with chronic phase advanced shifts markedly impaired estrous regularity. This suggests that internal desynchronization affects regular estrous functioning to a greater extent than alcohol consumption. Reports from female shift workers (Labyak et al., 2002; Cai et al., 2019), and studies in animals kept under artificial LD conditions support this view (Hardy, 1970; Yoshinaka et al., 2017). Future studies in ovariectomized individuals may further decipher the mutual interactions of chronodisruption, estrous cycle, sex hormone fluctuations, and alcohol consumption.

### Emotional behavior during alcohol abstinence

Extensive literature supports the association between circadian rhythm disruption and the emergence of anxiety and depression-like symptoms (Bedrosian and Nelson, 2017). Since negative emotional states may accelerate alcohol consumption and trigger a relapse, the association between circadian disruption and affective disorder is important in the context of the development and progression of aberrant alcohol-drinking behavior (Yang et al., 2018). In the present study, we used multiple behavioral measures to examine the emotional state changes of female rats during alcohol abstinence in the context of circadian disruption. As the time point for behavioral assessment is critical for the results and evaluation of behavior (Saré et al., 2021), we assessed anxiety-related behavior at two different points depending on the test (Nakano et al., 2016; Schoettner et al., 2022). Animals were tested at the same external time in correspondence to the LD conditions (Zeitgeber time). However, chronodisruption causes misalignments between internal and external circadian time. Thus, the internal circadian time of testing may vary across individuals within and between our experimental groups, despite testing at the same “external time.” Testing at various time points using the same animal may be beneficial in this regard, but difficult to achieve in common tests for anxiety-like behavior like the elevated-plus maze because of the well-described “one-time tolerance phenomenon” of those behavioral tests in rodents (File et al., 1990; Rodgers et al., 1992; Holmes and Rodgers, 1998, 1999). Alternatively, when measuring behavior using the internal time (e.g., onset of locomotor activity rhythm) as a reference, it may not correspond to the same external time across chronodisrupted animals, and the test may thus be affected by external conditions such as light. The uniform behavioral responses in our chronodisrupted animals, however, indicate that behavioral alterations occurred independently of external or internal time.

Reduced exploratory behavior and increased anxiety-like behavior in rats under alcohol withdrawal have been demonstrated in the past (Valdez et al., 2002; Overstreet et al., 2003; Rasmussen et al., 2006). In line with these studies, we observed reduced locomotion in the OFT under alcohol abstinence, demonstrating the deleterious effect of previous alcohol consumption on anxiety-related behavior. In contrast, anxiety-related behavior in the EPM female rats under conditions of alcohol abstinence was only observed in chronodisrupted animals. Previous work in male rats indicated that altered LD conditions alone induce anxiety-like behavior (Okuliarova et al., 2016; Horsey et al., 2020), which was not replicated in our study. The unequivocal response across anxiety tests indicated that the deleterious effects of chronodisruption and alcohol exposure and mood need to be considered for specific anxiety traits rather than generalized anxiety-like behavior. Moreover, sex, stress, or the use of running wheels may have mitigated the apparent effects of aberrant LD conditions on affective behaviors, depending on the behavioral test (Binder et al., 2004; Sandi et al., 2008; Novak et al., 2012; Bilu et al., 2022). Studies indicate that acute and chronic alcohol consumption modulates the expression and rhythmic pattern of circadian clock genes and affects various components of the stress hypothalamic-pituitary-adrenal (HPA)-axis (Perreau-Lenz and Spanagel, 2015). The combined aberrant effects of chronodisruption and alcohol consumption on the HPA axis may represent a central component in the control of mood-related alterations and should be investigated in more detail in future studies.

Increased reactive behavior during alcohol abstinence was not evident in the marble burying task. In our experiment, alcohol-experienced rats exhibited decreased burying behavior, whereas previous studies have shown that female rodents enhance marble-burying behavior during alcohol abstinence (Umathe et al., 2008; Leonardo Jimenez Chavez et al., 2020). As chronic phase shifts, alcohol binge drinking, and withdrawal have broad effects on cognition, memory function, and motivation, the ability to bury marbles might be even more compromised in the combination of all these (Stevenson et al., 2009; Ji et al., 2018). While the MBT is a widely accepted model to study anxiety and compulsive-like traits in rodents, findings depend strongly on contextually valid experimental design, and individual characteristics including cognitive function (de Brouwer et al., 2019).

Reduced sucrose consumption is often used as a measure of anhedonia to assess depressive-like traits in rodents (Liu et al., 2018). Studies exposing male rats to various LD schedules alone reported an increased depressive-like behavior associated with decreased sugar preference (Ben-Hamo et al., 2016; Horsey et al., 2020). We did not observe this behavior in alcohol-naïve female rats in our experiment. Factors including the experimental testing design for sugar preference (e.g., length and exposure of the sugar solution), as well as the female sex may be accountable for the different outcomes. Similarly, alcohol-experienced animals displayed no differences in sucrose preference. These results are in line with studies that reported a decreased or no change in sucrose preference during alcohol abstinence in female rodents (Pang et al., 2013; Metten et al., 2018; Li et al., 2019).

## Conclusion

Our study provides a foundation to understand how circadian desynchronization affects emotional behavior in female rats under alcohol abstinence. The female organism consists of a unique sex-dependent neurobiological, chronobiological, and hormonal setting, that distinctly reacts to chronic light-dark changes. In future studies, it will be important to distinguish between operational and acute sex-hormonal factors mediating the consequences of internal desynchronization and the effects of alcohol intake in females. A more comprehensive understanding of the role of sex-dependent circadian risk factors will be needed to provide sex-specific alcohol rehabilitation treatments incorporating affective disorders as an important factor in individuals performing under shiftwork conditions.

## Data availability statement

The original contributions presented in this study are included in the article/supplementary material, further inquiries can be directed to the corresponding authors.

## Ethics statement

The animal study was reviewed and approved by the Canadian Council on Animal Care and the Concordia University Animal Care Committee (certificate number: 30000256).

## Author contributions

CM and SA conceived and designed the experiments. CM conducted the experiments and assisted by KS. CM analyzed and interpreted the data with input from KS and SA. CM did the data visualization. CM wrote the manuscript and revised by SA and KS. SA supervised the project. All authors contributed to the article and approved the submitted version.

## Footnotes

1
www.circadian.org

## References

[B1] Abulseoud O. A. Karpyak V. M. Schneekloth T. Hall-Flavin D. K. Loukianova L. L. Geske J. R. (2013). A retrospective study of gender differences in depressive symptoms and risk of relapse in patients with alcohol dependence. *Am. J. Addict.* 22 437–442. 10.1111/j.1521-0391.2013.12021.x 23952888 PMC3748388

[B2] Ait-Daoud N. Blevins D. Khanna S. Sharma S. Holstege C. P. (2017). Women and addiction. *Psychiatr. Clin. North Am.* 40 285–297. 10.1016/j.psc.2017.01.005 28477653

[B3] An G. H. Chen X. W. Li C. Zhang L. Wei M. F. Chen J. J. (2020). Pathophysiological changes in female rats with estrous cycle disorder induced by long-term heat stress. *Biomed Res. Int.* 2020:4701563. 10.1155/2020/4701563 32685488 PMC7320282

[B4] Barbacka-Surowiak G. Surowiak J. Stokłosowa S. (2003). The involvement of suprachiasmatic nuclei in the regulation of estrous cycles in rodents. *Reprod. Biol.* 3 99–129. 14666136

[B5] Barko K. Shelton M. A. Seggio J. A. Logan R. W. (2019). Chapter 13 – Circadian rhythms and addiction. *Neural Mech. Addict.* 2019, 189–212. 10.1016/B978-0-12-812202-0.00013-0

[B6] Becker J. B. Koob G. F. (2016). Sex differences in animal models: Focus on addiction. *Pharmacol. Rev.* 68 242–263. 10.1124/pr.115.011163 26772794 PMC4813426

[B7] Bedrosian T. A. Nelson R. J. (2017). Timing of light exposure affects mood and brain circuits. *Transl. Psychiatry* 7:e1017. 10.1038/tp.2016.262 28140399 PMC5299389

[B8] Ben-Hamo M. Larson T. A. Duge L. S. Sikkema C. Wilkinson C. W. de la Iglesia H. O. (2016). Circadian forced desynchrony of the master clock leads to phenotypic manifestation of depression in rats. *eNeuro* 3:ENEURO.0237-16.2016. 10.1523/ENEURO.0237-16.2016 28090585 PMC5216685

[B9] Bilu C. Kronfeld-Schor N. Zimmet P. Einat H. (2022). Sex differences in the response to circadian disruption in diurnal sand rats. *Chronobiol. Int.* 39 169–185. 10.1080/07420528.2021.1989448 34711113

[B10] Binder E. Droste S. K. Ohl F. Reul J. M. H. M. (2004). Regular voluntary exercise reduces anxiety-related behaviour and impulsiveness in mice. *Behav. Brain Res.* 155 197–206. 10.1016/J.BBR.2004.04.017 15364478

[B11] Boulos Z. Rosenwasser A. M. (2004). “A chronobiological perspective on allostasis and its application to shift work,” in *Allostasis, homeostasis, and the costs of physiological adaptation*, ed. Jay S. (Cambridge: Cambridge University Press). 10.1017/CBO9781316257081.010

[B12] Brown L. A. Fisk A. S. Pothecary C. A. Peirson S. N. (2019). Telling the time with a broken clock: Quantifying circadian disruption in animal models. *Biology* 8:18. 10.3390/biology8010018 30901884 PMC6466320

[B13] Buhr T. J. Reed C. H. Shoeman A. Bauer E. E. Valentine R. J. Clark P. J. (2021). The influence of moderate physical activity on brain monoaminergic responses to binge-patterned alcohol ingestion in female mice. *Front. Behav. Neurosci.* 15:12. 10.3389/FNBEH.2021.639790/BIBTEX PMC7947191 33716684

[B14] Cai C. Vandermeer B. Khurana R. Nerenberg K. Featherstone R. Sebastianski M. (2019). The impact of occupational shift work and working hours during pregnancy on health outcomes: A systematic review and meta-analysis. *Am. J. Obstet. Gynecol.* 221 563–576. 10.1016/j.ajog.2019.06.051 31276631

[B15] Casiraghi L. P. Oda G. A. Chiesa J. J. Friesen W. O. Golombek D. A. (2012). Forced desynchronization of activity rhythms in a model of chronic jet lag in mice. *J. Biol. Rhythms* 27 59–69. 10.1177/0748730411429447 22306974

[B16] Clark J. W. Fixaris M. C. Belanger G. V. Rosenwasser A. M. (2007). Repeated light-dark phase shifts modulate voluntary ethanol intake in male and female high alcohol-drinking (HAD1) rats. *Alcohol. Clin. Exp. Res.* 31 1699–1706. 10.1111/j.1530-0277.2007.00476.x 17681032

[B17] Cora M. C. Kooistra L. Travlos G. (2015). Vaginal cytology of the laboratory rat and mouse: Review and criteria for the staging of the estrous cycle using stained vaginal smears. *Toxicol. Pathol.* 43 776–793. 10.1177/0192623315570339 25739587 PMC11504324

[B18] de Brouwer G. Fick A. Harvey B. H. Wolmarans D. W. (2019). A critical inquiry into marble-burying as a preclinical screening paradigm of relevance for anxiety and obsessive–compulsive disorder: Mapping the way forward. *Cogn. Affect. Behav. Neurosci.* 19 1–39. 10.3758/s13415-018-00653-4 30361863

[B19] de La Iglesia H. O. Cambras T. Schwartz W. J. Díez-Noguera A. (2004). Forced desynchronization of dual circadian oscillators within the rat suprachiasmatic nucleus. *Curr. Biol.* 14 796–800. 10.1016/j.cub.2004.04.034 15120072

[B20] Depoy L. M. McClung C. A. Logan R. W. (2017). Neural mechanisms of circadian regulation of natural and drug reward. *Neural Plast.* 2017:5720842. 10.1155/2017/5720842 29359051 PMC5735684

[B21] Eagle A. Mazei-Robison M. Robison A. (2016). Sucrose preference test to measure stress-induced anhedonia. *Bio Protoc.* 6:e1822. 10.21769/bioprotoc.1822

[B22] Ehringer M. A. Hoft N. R. Zunhammer M. (2009). Reduced alcohol consumption in mice with access to a running wheel. *Alcohol* 43 443–452. 10.1016/J.ALCOHOL.2009.06.003 19801274

[B23] Emanuele M. A. Wezeman F. Emanuele N. V. (2002). Alcohol’s effects on female reproductive function. *Alcohol Res. Health* 26 274–281. 12875037 PMC6676690

[B24] Erol A. Ho A. M. C. Winham S. J. Karpyak V. M. (2019). Sex hormones in alcohol consumption: A systematic review of evidence. *Addict. Biol.* 24 157–169. 10.1111/adb.12589 29280252 PMC6585852

[B25] File S. E. Mabbutt P. S. Hitchcott P. K. (1990). Characterisation of the phenomenon of “one-trial tolerance” to the anxiolytic effect of chlordiazepoxide in the elevated plus-maze. *Psychopharmacology* 102 98–101. 10.1007/BF02245751 1975449

[B26] Glantz M. D. Bharat C. Degenhardt L. Sampson N. A. Scott K. M. Lim C. C. W. (2020). The epidemiology of alcohol use disorders cross-nationally: Findings from the world mental health surveys. *Addict. Behav.* 102:106128. 10.1016/j.addbeh.2019.106128 31865172 PMC7416527

[B27] Hardy D. F. (1970). The effect of constant light on the estrous cycle and behavior of the female rat. *Physiol. Behav.* 5 421–425. 10.1016/0031-9384(70)90246-5 5535493

[B28] Heilig M. Egli M. Crabbe J. C. Becker H. C. (2010). Acute withdrawal, protracted abstinence and negative affect in alcoholism: Are they linked? *Addict. Biol.* 15 169–184. 10.1111/j.1369-1600.2009.00194.x 20148778 PMC3268458

[B29] Holmes A. Rodgers R. J. (1998). Responses of Swiss–webster mice to repeated plus-maze experience: Further evidence for a qualitative shift in emotional state? *Pharmacol. Biochem. Behav.* 60 473–488. 10.1016/S0091-3057(98)00008-2 9632231

[B30] Holmes A. Rodgers R. J. (1999). Influence of spatial and temporal manipulations on the anxiolytic efficacy of chlordiazepoxide in mice previously exposed to the elevated plus-maze. *Neurosci. Biobehav. Rev.* 23 971–980. 10.1016/S0149-7634(99)00030-5 10580311

[B31] Holzhauer C. G. Cucciare M. Epstein E. E. (2019). Sex and gender effects in recovery from alcohol use disorder. *Alcohol Res. Curr. Rev.* 40 1–19. 10.35946/arcr.v40.3.03 33224697 PMC7668196

[B32] Horsey E. A. Maletta T. Turner H. Cole C. Lehmann H. Fournier N. M. (2020). Chronic jet lag simulation decreases hippocampal neurogenesis and enhances depressive behaviors and cognitive deficits in adult male rats. *Front. Behav. Neurosci.* 13:272. 10.3389/fnbeh.2019.00272 31969809 PMC6960209

[B33] Ji Z. Yuan L. Lu X. Ding H. Luo J. Ke Z. J. (2018). Binge Alcohol exposure causes neurobehavioral deficits and GSK3β activation in the hippocampus of adolescent rats. *Sci. Rep.* 8:3088. 10.1038/s41598-018-21341-w 29449568 PMC5814471

[B34] Karpyak V. M. Biernacka J. M. Geske J. R. Abulseoud O. A. Brunner M. D. Chauhan M. (2016). Gender-specific effects of comorbid depression and anxiety on the propensity to drink in negative emotional states. *Addiction* 111 1366–1375. 10.1111/add.13386 27009547 PMC4940218

[B35] Koob G. F. (2009). Brain stress systems in the amygdala and addiction. *Brain Res.* 1293 61–75. 10.1016/J.BRAINRES.2009.03.038 19332030 PMC2774745

[B36] Koob G. F. Volkow N. D. (2016). Neurobiology of addiction: A neurocircuitry analysis. *Lancet Psychiatry* 3 760–773. 10.1016/S2215-0366(16)00104-8 27475769 PMC6135092

[B37] Kovanen L. Saarikoski S. T. Haukka J. Pirkola S. Aromaa A. Lönnqvist J. (2010). Circadian clock gene polymorphisms in alcohol use disorders and alcohol consumption. *Alcohol Alcohol.* 45 303–311. 10.1093/alcalc/agq035 20554694

[B38] Labyak S. Lava S. Turek F. Zee P. (2002). Effects of shiftwork on sleep and menstrual function in nurses. *Health Care Women Int.* 23 703–714. 10.1080/07399330290107449 12418990

[B39] Leise T. L. Harrington M. E. Molyneux P. C. Song I. Queenan H. Zimmerman E. (2013). Voluntary exercise can strengthen the circadian system in aged mice. *Age* 35 2137–2152. 10.1007/S11357-012-9502-Y/FIGURES/12 23340916 PMC3825002

[B40] Leonardo Jimenez Chavez C. Coelho M. A. Brewin L. W. Swauncy I. Tran T. Albanese T. (2020). Incubation of negative affect during protracted alcohol withdrawal is age-, but not sex-selective. *Brain Sci.* 10:405. 10.3390/brainsci10060405 32604806 PMC7348966

[B41] Li J. Chen P. Han X. Zuo W. Mei Q. Bian E. Y. (2019). Differences between male and female rats in alcohol drinking, negative affects and neuronal activity after acute and prolonged abstinence. *Int. J. Physiol. Pathophysiol. Pharmacol.* 11 163–176. 31523363 PMC6737432

[B42] Liu M. Y. Yin C. Y. Zhu L. J. Zhu X. H. Xu C. Luo C. X. (2018). Sucrose preference test for measurement of stress-induced anhedonia in mice. *Nat. Protoc.* 13 1686–1698. 10.1038/s41596-018-0011-z 29988104

[B43] Lyngsø J. Toft G. Høyer B. B. Guldbrandsen K. Olsen J. Ramlau-Hansen C. H. (2014). Moderate alcohol intake and menstrual cycle characteristics. *Hum. Reprod.* 29 351–358. 10.1093/humrep/det417 24287817

[B44] Lynskey M. T. (1998). The comorbidity of alcohol dependence and affective disorders: Treatment implications. *Drug Alcohol Depend.* 52 201–209. 10.1016/S0376-8716(98)00095-7 9839146

[B45] McHugh R. K. Votaw V. R. Sugarman D. E. Greenfield S. F. (2018). Sex and gender differences in substance use disorders. *Clin. Psychol. Rev.* 66 12–23. 10.1016/j.cpr.2017.10.012 29174306 PMC5945349

[B46] McKetta S. Keyes K. M. (2019). Heavy and binge alcohol drinking and parenting status in the United States from 2006 to 2018: An analysis of nationally representative cross-sectional surveys. *PLoS Med.* 16:e1002954. 10.1371/journal.pmed.1002954 31770389 PMC6879113

[B47] Metten P. Schlumbohm J. P. Huang L. C. Greenberg G. D. Hack W. R. Spence S. E. (2018). An alcohol withdrawal test battery measuring multiple behavioral symptoms in mice. *Alcohol* 68 19–35. 10.1016/j.alcohol.2017.08.014 29427828 PMC5839916

[B48] Mill D. J. Bito-Onon J. J. Simms J. A. Li R. Bartlett S. E. (2013). Fischer rats consume 20% ethanol in a long-term intermittent-access two-bottle-choice paradigm. *PLoS One* 8:e79824. 10.1371/journal.pone.0079824 24244567 PMC3828209

[B49] Nakano J. J. Shimizu K. Shimba S. Fukada Y. (2016). SCOP/PHLPP1β in the basolateral amygdala regulates circadian expression of mouse anxiety-like behavior. *Sci. Rep.* 6:33500. 10.1038/srep33500 27640726 PMC5027591

[B50] Novak C. M. Burghardt P. R. Levine J. A. (2012). The use of a running wheel to measure activity in rodents: Relationship to energy balance, general activity, and reward. *Neurosci. Biobehav. Rev.* 36 1001–1014. 10.1016/J.NEUBIOREV.2011.12.012 22230703 PMC4455940

[B51] Okuliarova M. Molcan L. Zeman M. (2016). Decreased emotional reactivity of rats exposed to repeated phase shifts of light-dark cycle. *Physiol. Behav.* 156 16–23. 10.1016/j.physbeh.2016.01.003 26773465

[B52] Overstreet D. H. Knapp D. J. Moy S. S. Breese G. R. (2003). A 5-HT1A agonist and a 5-HT2c antagonist reduce social interaction deficit induced by multiple ethanol withdrawals in rats. *Psychopharmacology* 167 344–352. 10.1007/S00213-003-1425-Y 12677355 PMC2865243

[B53] Ozburn A. R. Falcon E. Mukherjee S. Gillman A. Arey R. Spencer S. (2013). The role of clock in ethanol-related behaviors. *Neuropsychopharmacology* 38 2393–2400. 10.1038/npp.2013.138 23722243 PMC3799058

[B54] Ozburn A. R. Harris R. A. Blednov Y. A. (2008). Wheel running, voluntary ethanol consumption, and hedonic substitution. *Alcohol* 42 417–424. 10.1016/J.ALCOHOL.2008.04.006 18579336 PMC2575879

[B55] Palma-Álvarez R. F. Rodríguez-Cintas L. Abad A. C. Sorribes M. Ros-Cucurull E. Robles-Martínez M. (2019). Mood disorders and severity of addiction in alcohol-dependent patients could be mediated by sex differences. *Front. Psychiatry* 10:343. 10.3389/fpsyt.2019.00343 31214056 PMC6554686

[B56] Pang T. Y. Renoir T. Du X. Lawrence A. J. Hannan A. J. (2013). Depression-related behaviours displayed by female C57BL/6J mice during abstinence from chronic ethanol consumption are rescued by wheel-running. *Eur. J. Neurosci.* 37 1803–1810. 10.1111/ejn.12195 23551162

[B57] Parekh P. K. Ozburn A. R. McClung C. A. (2015). Circadian clock genes: Effects on dopamine, reward and addiction. *Alcohol* 49 341–349. 10.1016/j.alcohol.2014.09.034 25641765 PMC4457686

[B58] Partonen T. (2012). Clock gene variants in mood and anxiety disorders. *J. Neural Transm.* 119 1133–1145. 10.1007/s00702-012-0810-2 22538398

[B59] Partonen T. (2015). Clock genes in human alcohol abuse and comorbid conditions. *Alcohol* 49 359–365. 10.1016/j.alcohol.2014.08.013 25677407

[B60] Peltier M. R. Verplaetse T. L. Mineur Y. S. Petrakis I. L. Cosgrove K. P. Picciotto M. R. (2019). Sex differences in stress-related alcohol use. *Neurobiol. Stress* 10:100149. 10.1016/j.ynstr.2019.100149 30949562 PMC6430711

[B61] Perreau-Lenz S. Spanagel R. (2015). Clock genes×stress×reward interactions in alcohol and substance use disorders. *Alcohol* 49 351–357. 10.1016/j.alcohol.2015.04.003 25943583 PMC4457607

[B62] Piza-Palma C. Barfield E. T. Brown J. A. Hubka J. C. Lusk C. Schonhar C. A. (2014). Oral self-administration of EtOH: Sex-dependent modulation by running wheel access in C57BL/6J mice. *Alcohol. Clin. Exp. Res.* 38 2387–2395. 10.1111/ACER.12519 25257288 PMC5495182

[B63] Priddy B. M. Carmack S. A. Thomas L. C. Vendruscolo J. C. M. Koob G. F. Vendruscolo L. F. (2017). Sex, strain, and estrous cycle influences on alcohol drinking in rats. *Pharmacol. Biochem. Behav.* 152 61–67. 10.1016/j.pbb.2016.08.001 27498303 PMC5755698

[B64] Prut L. Belzung C. (2003). The open field as a paradigm to measure the effects of drugs on anxiety-like behaviors: A review. *Eur. J. Pharmacol.* 463 3–33. 10.1016/S0014-2999(03)01272-X 12600700

[B65] Rasmussen D. D. Wilkinson C. W. Raskind M. A. (2006). Chronic daily ethanol and withdrawal: 6. Effects on rat sympathoadrenal activity during “abstinence”. *Alcohol* 38 173–177. 10.1016/j.alcohol.2006.06.007 16905443 PMC1839872

[B66] Refinetti R. (1992). Analysis of the circadian rhythm of body temperature. *Behav. Res. Methods Instrum. Comput.* 24 28–36.

[B67] Rehm J. Mathers C. Popova S. Thavorncharoensap M. Teerawattananon Y. Patra J. (2009). Global burden of disease and injury and economic cost attributable to alcohol use and alcohol-use disorders. *Lancet* 373 2223–2233. 10.1016/S0140-6736(09)60746-7 19560604

[B68] Reséndiz-Flores M. Escobar C. (2019). Circadian disruption favors alcohol consumption and differential ΔFosB accumulation in corticolimbic structures. *Addict. Biol.* 24 1179–1190. 10.1111/adb.12674 30295391

[B69] Richter K. Peter L. Rodenbeck A. Weess H. G. Riedel-Heller S. G. Hillemacher T. (2021). Shiftwork and alcohol consumption: A systematic review of the literature. *Eur. Addict. Res.* 27 9–15. 10.1159/000507573 32454482

[B70] Rizk A. A. Jenkins B. W. Al-Sabagh Y. Hamidullah S. Reitz C. J. Rasouli M. (2022). The impact of sex, circadian disruption, and the clockΔ19/Δ19 genotype on alcohol drinking in mice. *Genes* 13:701. 10.3390/genes13040701 35456507 PMC9031797

[B71] Rodgers R. J. Lee C. Shepherd J. K. (1992). Effects of diazepam on behavioural and antinociceptive responses to the elevated plus-maze in male mice depend upon treatment regimen and prior maze experience. *Psychopharmacology* 106 102–110. 10.1007/BF02253596 1738787

[B72] Rosenwasser A. M. Clark J. W. Fixaris M. C. Belanger G. V. Foster J. A. (2010). Effects of repeated light-dark phase shifts on voluntary ethanol and water intake in male and female Fischer and Lewis rats. *Alcohol* 44 229–237. 10.1016/j.alcohol.2010.03.002 20488643

[B73] Rosenwasser A. M. Fixaris M. C. McCulley W. D. (2015). Photoperiodic modulation of voluntary ethanol intake in C57BL/6 mice. *Physiol. Behav.* 147 342–347. 10.1016/j.physbeh.2015.05.011 25992479

[B74] Rosenwasser A. M. McCulley W. D. Fecteau M. (2014). Circadian activity rhythms and voluntary ethanol intake in male and female ethanol-preferring rats: Effects of long-term ethanol access. *Alcohol* 48 647–655. 10.1016/j.alcohol.2014.07.010 25281289 PMC4250354

[B75] Sandi C. Cordero M. I. Ugolini A. Varea E. Caberlotto L. Large C. H. (2008). Chronic stress-induced alterations in amygdala responsiveness and behavior – modulation by trait anxiety and corticotropin-releasing factor systems. *Eur. J. Neurosci.* 28 1836–1848. 10.1111/J.1460-9568.2008.06451.X 18973598

[B76] Saré R. M. Lemons A. Smith C. B. Smith-Hicks C. (2021). Brain sciences review behavior testing in rodents: Highlighting potential confounds affecting variability and reproducibility section on neuroadaptation and protein metabolism. *Brain Sci.* 11:522. 10.3390/brainsci11040522 33924037 PMC8073298

[B77] Satta R. Hilderbrand E. R. Lasek A. W. (2018). Ovarian hormones contribute to high levels of binge-like drinking by female mice. *Alcohol. Clin. Exp. Res.* 42 286–294. 10.1111/acer.13571 29205408 PMC5785425

[B78] Schneider T. Popik P. (2007). Attenuation of estrous cycle-dependent marble burying in female rats by acute treatment with progesterone and antidepressants. *Psychoneuroendocrinology* 32 651–659. 10.1016/j.psyneuen.2007.04.003 17561352

[B79] Schoettner K. Alonso M. Button M. Goldfarb C. Herrera J. Quteishat N. (2022). Characterization of affective behaviors and motor functions in mice with a striatal-specific deletion of bmal1 and per2. *Front. Physiol.* 13:922080. 10.3389/fphys.2022.922080 35755440 PMC9216244

[B80] Sciarra F. Franceschini E. Campolo F. Gianfrilli D. Pallotti F. Paoli D. (2020). Disruption of circadian rhythms: A crucial factor in the etiology of infertility. *Int. J. Mol. Sci.* 21:3943. 10.3390/ijms21113943 32486326 PMC7312974

[B81] Simms J. A. Steensland P. Medina B. Abernathy K. E. Chandler L. J. Wise R. (2008). Intermittent access to 20% ethanol induces high ethanol consumption in Long-Evans and Wistar rats. *Alcohol. Clin. Exp. Res.* 32 1816–1823. 10.1111/j.1530-0277.2008.00753.x 18671810 PMC3151464

[B82] Sleipness E. P. Sorg B. A. Jansen H. T. (2007). Diurnal differences in dopamine transporter and tyrosine hydroxylase levels in rat brain: Dependence on the suprachiasmatic nucleus. *Brain Res.* 1129 34–42. 10.1016/J.BRAINRES.2006.10.063 17156761

[B83] Sokolove P. G. Bushell W. N. (1978). The chi square periodogram: Its utility for analysis of circadian rhythms. *J. Theor. Biol.* 72 131–160. 10.1016/0022-5193(78)90022-X 566361

[B84] Spanagel R. Pendyala G. Abarca C. Zghoul T. Sanchis-Segura C. Magnone M. C. (2005). The clock gene Per2 influences the glutamatergic system and modulates alcohol consumption. *Nat. Med.* 11 35–42. 10.1038/nm1163 15608650

[B85] Statistics Canada (2013). *Canadian community health survey: Mental health. The daily.* Ottawa, ON: Statistics Canada.

[B86] Stevenson J. R. Schroeder J. P. Nixon K. Besheer J. Crews F. T. Hodge C. W. (2009). Abstinence following alcohol drinking produces depression-like behavior and reduced hippocampal neurogenesis in mice. *Neuropsychopharmacology* 34 1209–1222. 10.1038/npp.2008.90 18563059 PMC2844649

[B87] Tamura E. K. Oliveira-Silva K. S. Ferreira-Moraes F. A. Marinho E. A. V. Guerrero-Vargas N. N. (2021). Circadian rhythms and substance use disorders: A bidirectional relationship. *Pharmacol. Biochem. Behav.* 201:173105. 10.1016/j.pbb.2021.173105 33444601

[B88] Umathe S. Bhutada P. Dixit P. Shende V. (2008). Increased marble-burying behavior in ethanol-withdrawal state: Modulation by gonadotropin-releasing hormone agonist. *Eur. J. Pharmacol.* 587 175–180. 10.1016/j.ejphar.2008.03.035 18448097

[B89] Valdez G. R. Roberts A. J. Chan K. Davis H. Brennan M. Zorrilla E. P. (2002). Increased ethanol self-administration and anxiety-like behavior during acute ethanol withdrawal and protracted abstinence: Regulation by corticotropin-releasing factor. *Alcohol. Clin. Exp. Res.* 26 1494–1501. 10.1097/01.ALC.0000033120.51856.F0 12394282

[B90] Vetter C. (2018). Circadian disruption: What do we actually mean? *Eur. J. Neurosci.* 51 531–550. 10.1111/ejn.14255 30402904 PMC6504624

[B91] Walf A. A. Frye C. A. (2007). The use of the elevated plus maze as an assay of anxiety-related behavior in rodents. *Nat. Protoc.* 2 322–328. 10.1038/nprot.2007.44 17406592 PMC3623971

[B92] Weinert D. Gubin D. (2022). The impact of physical activity on the circadian system: Benefits for health, performance and Wellbeing. *Appl. Sci.* 12:9220. 10.3390/APP12189220

[B93] Weinert D. Schöttner K. Müller L. Wienke A. (2016). Intensive voluntary wheel running may restore circadian activity rhythms and improves the impaired cognitive performance of arrhythmic djungarian hamsters. *Chronobiol. Int.* 33 1161–1170. 10.1080/07420528.2016.1205083 27459238

[B94] Wise R. A. (1973). Voluntary ethanol intake in rats following exposure to ethanol on various schedules. *Psychopharmacologia* 29 203–210. 10.1007/BF00414034 4702273

[B95] Wollnik F. Turek F. W. (1988). Estrous correlated modulations of circadian and ultradian wheel-running activity rhythms in LEW/Ztm rats. *Physiol. Behav.* 43 389–396. 10.1016/0031-9384(88)90204-1 3262879

[B96] Yang P. Tao R. He C. Liu S. Wang Y. Zhang X. (2018). The risk factors of the alcohol use disorders-through review of its comorbidities. *Front. Neurosci.* 12:303. 10.3389/fnins.2018.00303 29867316 PMC5958183

[B97] Yoshinaka K. Yamaguchi A. Matsumura R. Node K. Tokuda I. Akashi M. (2017). Effect of different light–dark schedules on estrous cycle in mice, and implications for mitigating the adverse impact of night work. *Genes Cells* 22 876–884. 10.1111/gtc.12522 28884885

[B98] Zucker I. Beery A. K. (2010). Males still dominate animal studies. *Nature* 465:690. 10.1038/465690a 20535186

